# Supplementary material for: Periaqueductal gray neurons encode the sequential motor program in hunting behavior of mice
Source: Nat Commun. 2021 Nov 11;12:6523. doi: 10.1038/s41467-021-26852-1 (PMC8586038; doi:10.1038/s41467-021-26852-1)
Supplement: Supplementary file 1 — Supplementary information [file 41467_2021_26852_MOESM1_ESM.pdf]

Supplementary Information

Periaqueductal Gray Neurons Encode the Sequential Motor Program in Hunting Behavior of mice

Hong Yu[1, 2, 3, 8], Xinkuan Xiang[1, 2, 8], Zongming Chen[4, 8], Xu Wang[1, 2], Jiaqi Dai[1, 2], Xinxin Wang[1, 2], Pengcheng Huang[1, 2], Zheng-dong Zhao[5], Wei L. Shen[4 *] & Haohong Li[6, 7 *]

[1]Britton Chance Center for Biomedical Photonics, Wuhan National Laboratory for Optoelectronics, Huazhong University of Science and Technology, Wuhan, Hubei 430074, China

[2]MoE Key Laboratory for Biomedical Photonics, Collaborative Innovation Center for Biomedical Engineering, School of Engineering Sciences, Huazhong University of Science and Technology, Wuhan, Hubei 430074, China

[3]College of Basic Medicine, Hubei University of Medicine, Shiyan, Hubei 442000, China

[4]School of Life Science and Technology & Shanghai Institute of Advanced Immunochemical Studies, Shanghaitech University, Shanghai, 201210, China

[5]Program in Cellular and Molecular Medicine, Boston Children's Hospital, Boston, Massachusetts 02115, USA

[6]Affiliated Mental Health Centre & Hangzhou Seventh People's Hospital, Zhejiang University School of Medicine, Hangzhou, Zhejiang 310013, China

[7]The MOE Frontier Research Center of Brain & Brain-machine Integration, Zhejiang University School of Brain Science and Brain Medicine, Hangzhou, Zhejiang 310058, China

[8]These authors contributed equally: Hong Yu, Xinkuan Xiang, Zongming Chen

*e-mail: shenwei@shanghaitech.edu.cn (W.L.S.); hhli_27@zju.edu.cn (H.H.L.)

Supplementary Fig. 1: Electrode and fiber locations.

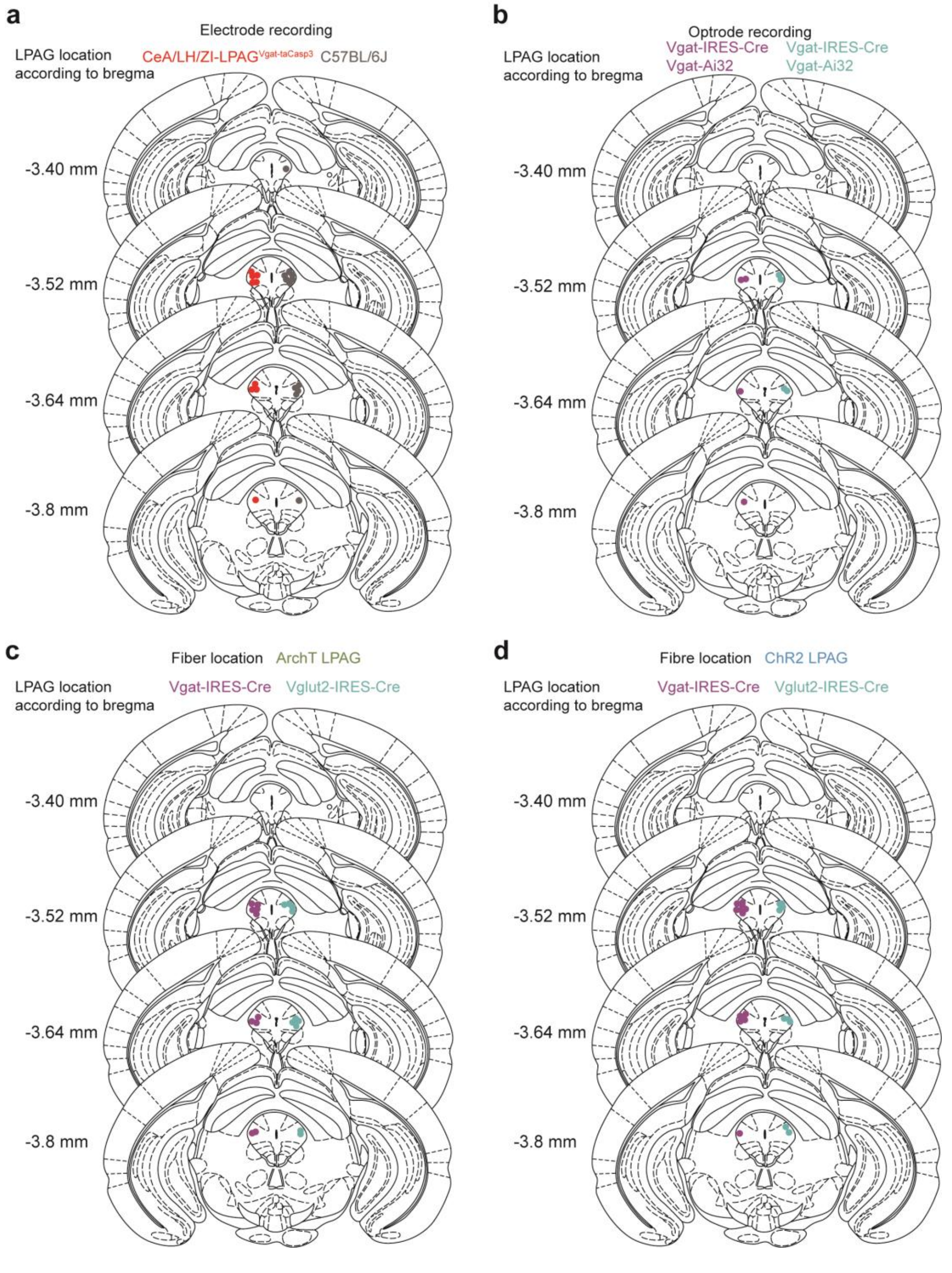

a, Electrode locations for *in vivo* single-uint recordings in consecutive coronal brain slices. **b**, Optrode locations in Vgat-IRES-Cre and Vgat-Ai32 mice, or in Vglut2-IRES-Cre and Vglut2-Ai32 mice. **c**, Fiber locations in Vgat-IRES-Cre and Vglut2-IRES-Cre mice of ArchT groups. **d**, Fiber locations in Vgat-IRES-Cre and Vglut2-IRES-Cre mice of ChR2 groups.

Supplementary Fig. 2: Phenotypes of LPAG neurons during hunting.

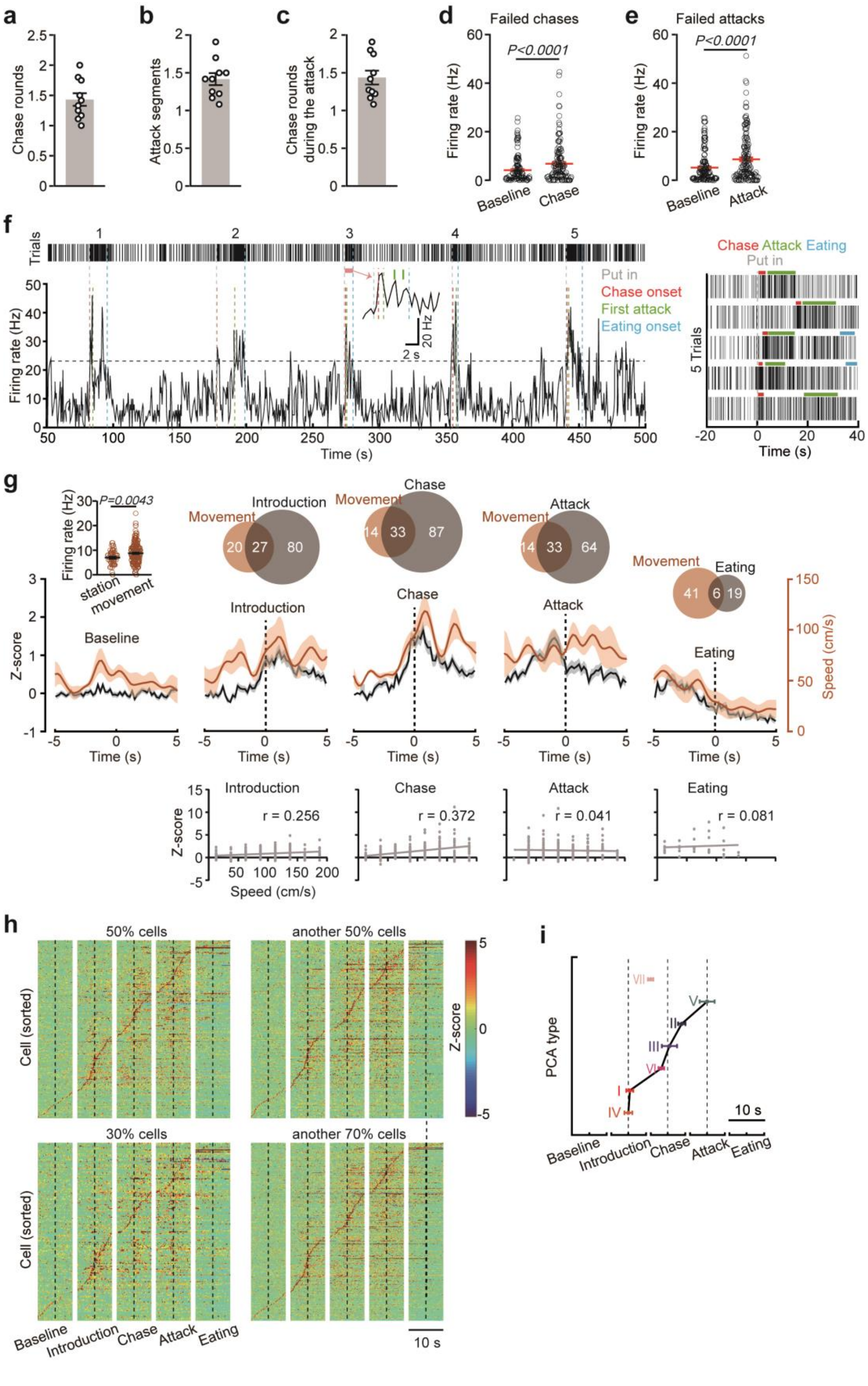

a, The number of chase rounds (n = 10 mice). **b**, The number of attack segments (n = 10 mice). **c**, The number of chase rounds during the attack phase (n = 10 mice). **d**, Firing rates of LPAG neurons during failed chases (n = 151 neurons, two-sided Wilcoxon signed-rank test). **e**, Firing rates of LPAG neurons during failed attacks (n = 155 neurons, two-sided Wilcoxon signed-rank test,). **f**, A typical neuronal activity trace (5 trials/session) during different predatory phases. The black dotted line represents a 95% confidence interval for firing rates of the baseline. The grey dotted lines represent the timepoints of put-in of crickets. The red dotted lines in the left represent the timepoints of chase onset and red lines in the right represent chase phases. The green dotted lines in the left represent the timepoints of first attack and green lines in the right represent attack phases. The blue dotted lines in the left represent the timepoints of eating onset and blue lines in the right represent eating phases. **g**, Top, normalized firing rates (black) and velocity curves (orange) during each hunting phase. Movement cells were defined that average firing rates during the movement were significantly higher than that of the station (two-tailed unpaired t-test). Pie charts represent the overlap between movement cells and introduction cells, chase cells, attack cells or eating cells, respectively. Dotted lines aligned the timepoints of put-in of crickets, chase onset, first attack and eating onset, respectively. Bottom, the activity-speed correlation (P = 0.008 during the introduction, P < 0.0001 during the chase, P = 0.5973 during the attack, P = 0.6652 during the eating, Pearson's r). **h**, Top, normalized responses of 50% (left) and another 50% (right) of recorded neurons during predatory hunting, sorted by their peak of responses. Bottom, normalized responses of 30% (left) and another 70% (right) of recorded neurons during predatory hunting, sorted by their peak of responses. Dotted lines represent the timepoints of put-in of crickets, chase onset, first attack, and eating onset, respectively. **i**, The average peak response time of PCA-clustered cells during five predatory phases. Dotted lines represent the timepoints of put-in of crickets, chase onset and first attack, respectively. Data are presented as the mean ± SEM. P values are indicated in all panels. Source data are provided as a Source Data File.

Supplementary Fig. 3: Specific behavioral details in the introduction phase.

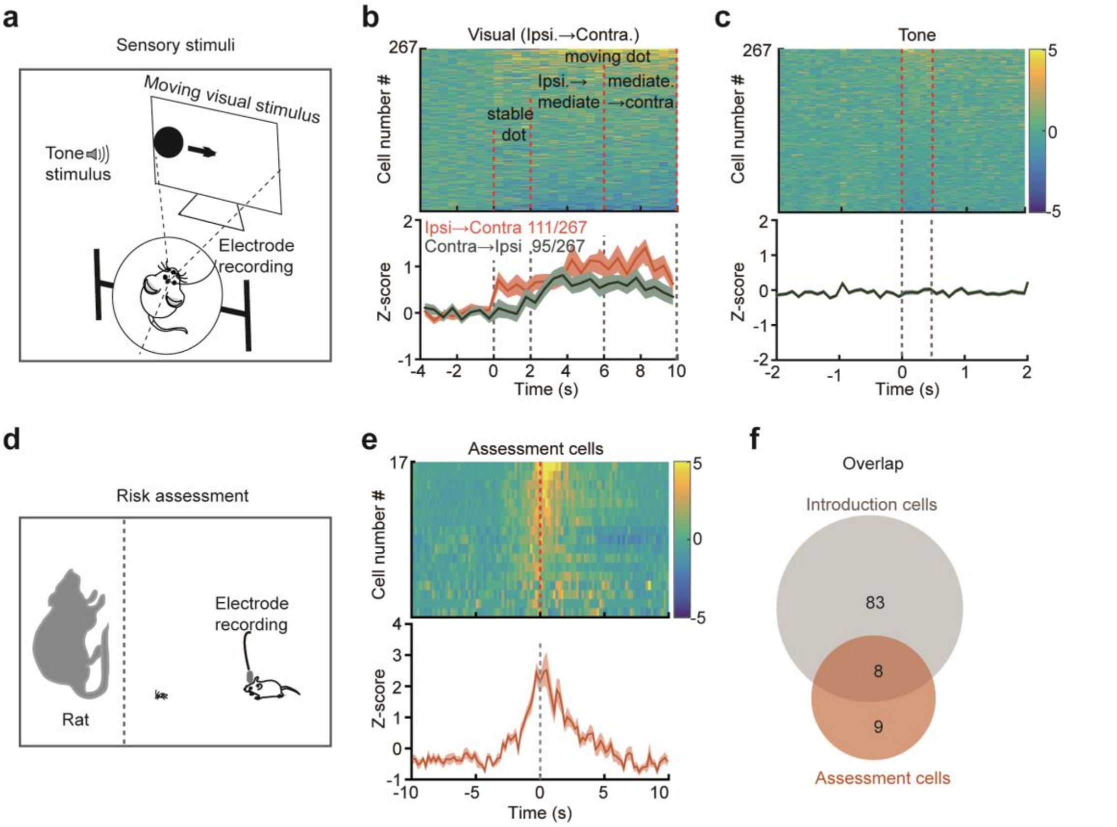

a, Scheme of head-fixed experimental device for single-unit recording during visual and auditory stimuli. **b**, Top, heatmap of moving dot-induced neuronal activities by the black dot from ipsi. to contra. (n = 267 units). Bottom, normalized firing rates of visual-excited neurons by the moving dot in two directions. The number of visual-excited neurons from ipsi. to contra. was 111, while from ipsi. to contra. was 95. The dotted lines respectively aligned to the time point of 2 s-duration stable dot, 4 s-duration moving dot, 4 s-duration from moving to stable dot. Shaded areas represent SEM. **c**, Top, heatmap of tone-induced responses (n = 267 units). Bottom, normalized firing rates of 267 recorded neurons. Tone was delivered between the two dotted lines (60 dB, 0.5 s). Shaded area represent error bands. **d**, Scheme of single-unit recording during the introduction phase and the risk assessment. **e**, Top, heatmap of assessment-excited neuronal activities (n = 17 units). Bottom, normalized firing rates of assessment cells. The dotted line aligned to the assessment onset. Shaded areas represent SEM. **f**, The overlap between introduction cells and assessment cells.

Supplementary Fig. 4: Activities of units recorded in the LPAG during different targets.

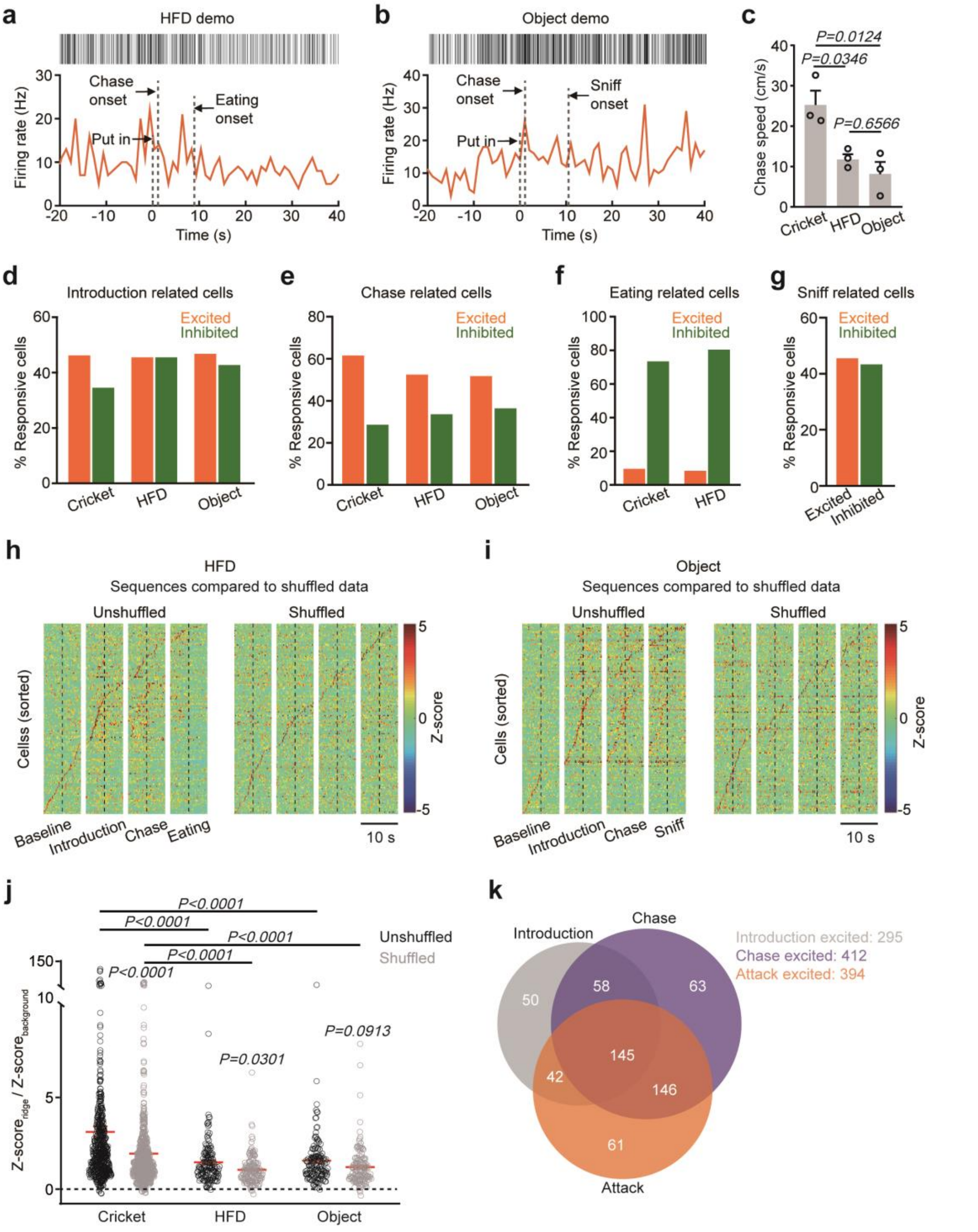

a, A typical neuronal activity changes during the interaction with HFD. **b**, A typical neuronal activity changes during the interaction with objects. **c**, Chase speed during running to different targets (n = 3 mice, one-way ANOVA, Tukey's multiple comparisons test). **d**, Percentage of responses to crickets, HFD, and objects during the introduction (crickets excited: 67 of 145, inhibited: 50 of 145; HFD excited: 64 of 145, inhibited: 63 of 145; objects excited: 68 of 145, inhibited: 62 of 145). **e**, Percentage of responses to crickets, HFD, and objects during the chase (crickets excited: 88 of 145, inhibited: 42 of 145; HFD excited: 76 of 145, inhibited: 48 of 145; objects excited: 76 of 145,

inhibited: 52 of 145). **f**, Percentage of responses to crickets and HFD during the eating (crickets excited: 13 of 145, inhibited: 106 of 145; HFD excited: 13 of 145, inhibited: 113 of 145). **g**, Percentage of responses to sniffing objects (excited: 67 of 145, inhibited: 62 of 145). **h**, Left, normalized responses of all recorded neurons during interaction to HFD, sorted by their peak of responses. Right, the same neurons as in the left panel, except with shuffled datasets (based on 1000 shuffles). Dotted lines represent the timepoints of put-in of HFD, chase onset, and eating onset, respectively. **i**, Left, normalized responses of all recorded neurons during interaction to objects, sorted by their peak of responses. Right, the same neurons as in the left panel, except with shuffled datasets (based on 1000 shuffles). Dotted lines represent the timepoints of put-in of objects, chase onset, and sniff onset, respectively. **j,** Ridge-to-background Z-score ratio for the plots from g and h. Comparison of the selectivity between unshuffled and shuffled data during predatory hunting (n = 618 neurons), interaction to HFD (n = 145 neurons), and interaction to objects (n = 145 neurons) (Kruskal-Wallis test). The ridge was defined as the mean z-score in 5 bins surrounding the peak value, and the background was defined as the mean z-score in all data points. The ridge-to-background ratio provides a measure of how selective the activity of the cell was during hunting. **k**, The overlap between introduction, chase and attack excited cells. Data are presented as the mean ± SEM. *P* values are indicated in all panels. Source data are provided as a Source Data File.

Supplementary Fig. 5: Optogenetic identification of LPAG^{Vgat} and LPAG^{Vglut2} neurons during predatory hunting.

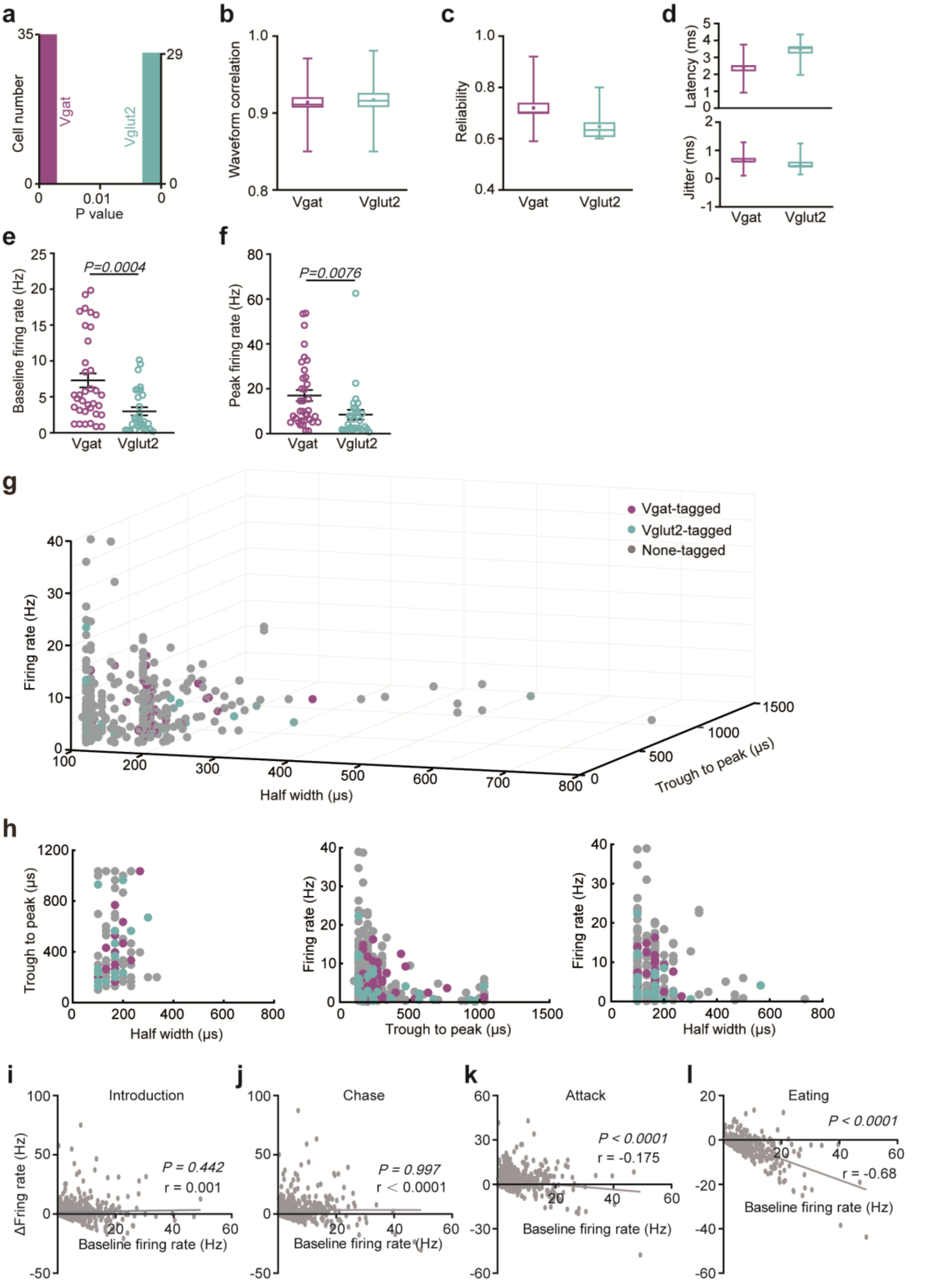

a, Histogram of the stimulus-associated spike latency test (SALT) for optical tagging 35 LPAG^{Vgat} neurons and 29 LPAG^{Vglut2} neurons. One-sided SALT, *P* values < 0.01. **b**, Correlation coefficient between spontaneous and light-evoked waveforms of all tagged LPAG^{Vgat} (light purple, n = 35) and LPAG^{Vglut2} (light blue, n = 29) neurons. **c**, Reliability of laser-evoked spikes in all identified

LPAG^{Vgat} (light purple, n = 35) and LPAG^{Vglut2} (light blue, n = 29) neurons. **d**, Box plots for all tagged LPAG^{Vgat} (light purple, n = 35) and LPAG^{Vglut2} (light blue, n = 29) neurons showed low light-evoked first-spike latency (top) and small jitter (bottom). Box plots in **b-d**: top line, maxima; bottom line, minima; center line, median; box limits, upper and lower quartiles; whiskers, 1.5 × interquartile range; points, values. **e**, Baseline firing rates of all tagged LPAG^{Vgat} (light purple, n = 35) and LPAG^{Vglut2} (light blue, n = 29) neurons (Mann Whitney U test). **f**, Peak firing rates of all tagged LPAG^{Vgat} (light purple, n = 35) and LPAG^{Vglut2} (light blue, n = 29) neurons (Mann Whitney U test). **g**, The distribution of LPAG neurons, tagged LPAG^{Vgat} neurons and tagged LPAG^{Vglut2} neurons based on firing rate, half-width and trough-to-peak. **h**, Left, the distribution of LPAG neurons, tagged LPAG^{Vgat} neurons and tagged LPAG^{Vglut2} neurons based on half-width and trough-to-peak. Middle, the distribution of LPAG neurons, tagged LPAG^{Vgat} neurons and tagged LPAG^{Vglut2} neurons based on trough-to-peak and firing rate. Right, the distribution of LPAG neurons, tagged LPAG^{Vgat} neurons and tagged LPAG^{Vglut2} neurons based on half-width and firing rate. **i**, Correlation between the change in firing rates during the introduction phase and the baseline firing rates (P = 0.442, Pearson's r). **j**, Correlation between the change in firing rates during the chase phase and the baseline firing rates (P = 0.997, Pearson's r). **k**, Correlation between the change in firing rates during the attack phase and the baseline firing rates ($P < 0.0001$, Pearson's r). **l**, Correlation between the change in firing rates during the eating phase and the baseline firing rates ($P < 0.0001$, Pearson's r). Data are presented as the mean ± SEM. P values are indicated in all panels. Source data are provided as a Source Data File.

Supplementary Fig. 6: Phenotypes of photoinhibition of LPAG neurons.

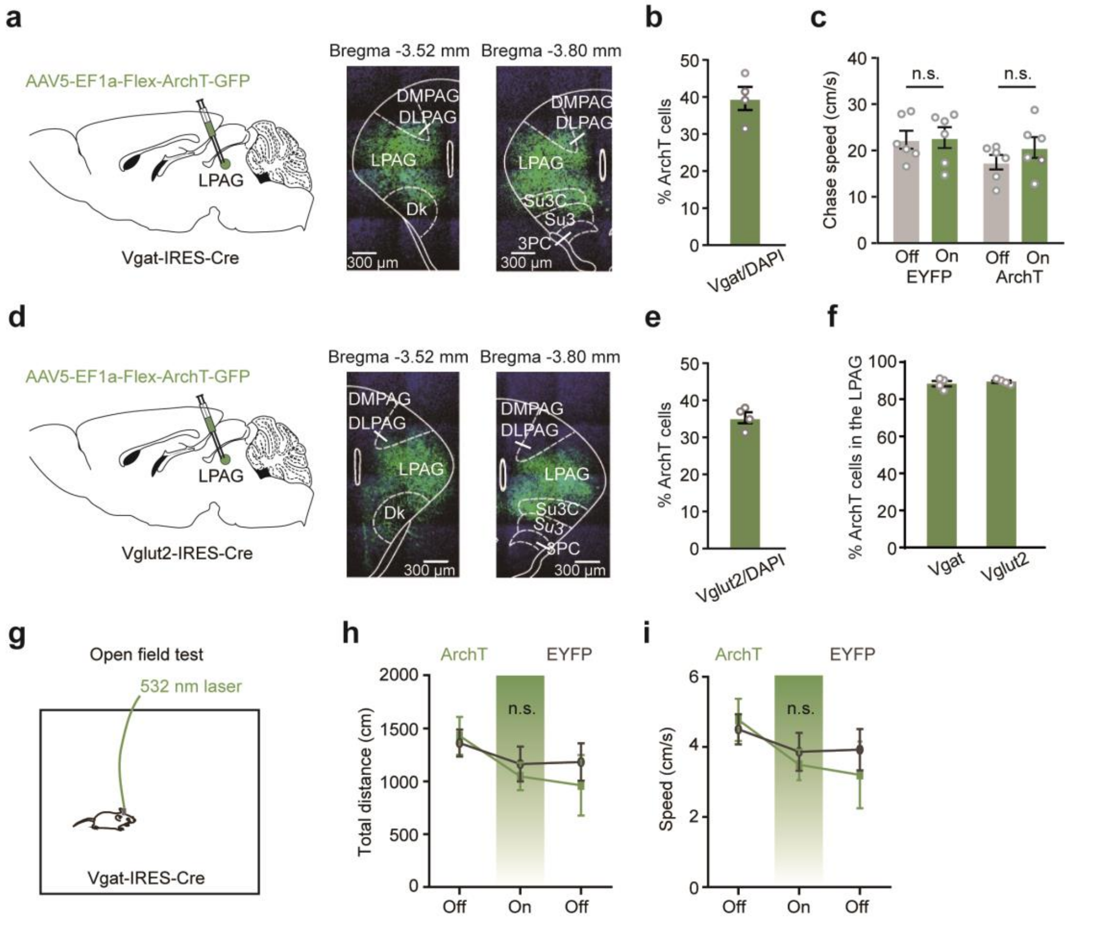

a, Left, scheme for injections of Vgat-IRES-Cre mice. Right, histology of injection sites in the LPAG. Scale bars, 300 μm. **b**, Percentage of $LPAG^{Vgat\text{-}ArchT}/LPAG^{DAPI}$ neurons (n = 4 mice). **c**, Speed during photogenetic inhibition of $LPAG^{Vgat}$ neurons in the chase phase (n = 6 mice for each group, two-tailed paired t-test, $P > 0.05$, n.s., no significance). **d**, Left, scheme for injections of Vglut2-IRES-Cre mice. Right, histology of injection sites in the LPAG. Scale bars, 300 μm. **e**, Percentage of $LPAG^{Vglut2\text{-}ArchT}/LPAG^{DAPI}$ neurons (n = 4 mice). **f**, Percentage of Vgat+ and Vglut2+ neurons by ArchT infected spread localized to the LPAG (n = 4 mice for each group). **g-i**, The open field test. Scheme (**g**), total distance (**h**), average velocity (**i**) before, during and after bilateral inhibition of $LPAG^{Vgat}$ neurons (n = 6 mice for each group, two-way repeated measures ANOVA, Bonferroni's multiple comparisons test, $P > 0.05$, n.s., no significance). Laser pattern: 532 nm, 12 mW, 30-s on/30-s off cycles. Data are presented as the mean ± SEM. Source data are provided as a Source Data File.

Supplementary Fig. 7: LPAG^{Vgat} neurons induce positive motivation.

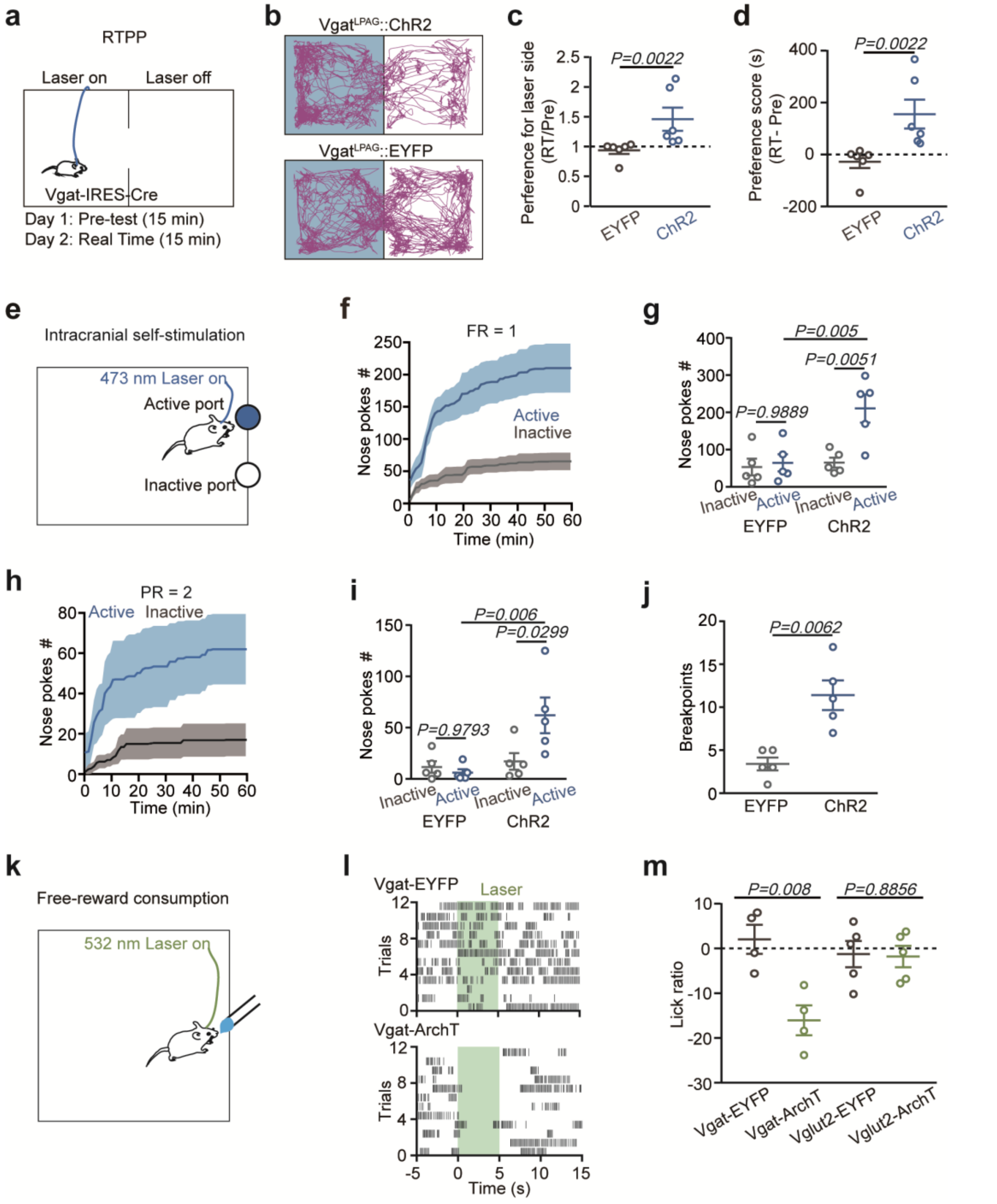

a, Diagram of real-time place preference (RTPP) assay. **b**, An example of traces of LPAG$^{Vgat\text{-}ChR2/EYFP}$ mice. **c**, Real time (RT) / pre-test ratios of time spent in laser-coupled chamber (two-sided Mann Whitney U test). LPAG$^{Vgat\text{-}EYFP}$ (gray), n = 6; LPAG$^{Vgat\text{-}ChR2}$ (blue), n = 6. **d**, Preference scores of indicated genotypes. The preference score is the time spent in the laser-coupled chamber, subtracted by the time spent during the pre-test (two-sided Mann Whitney U test). LPAG$^{Vgat\text{-}EYFP}$ (gray), n = 6; LPAG$^{Vgat\text{-}ChR2}$ (blue), n = 6. **e**, Scheme of the intracranial self-stimulation paradigm. A 20-Hz optogenetic self-stimulation was given in the active port. **f**, Cumulative nose pokes under

the fixed ratio =1 (FR1) task, while mice needed to poke once to receive a 3-s laser stimulation. **g**, The total number of nose pokes under the FR1 task (n = 5 mice for each group, two-way repeated-measures ANOVA, Tukey's multiple comparisons test). **h**, Cumulative nose pokes under the progressive ratio = 2 (PR2) task, while mice needed to increase poking by two to receive a 3-s laser stimulation. **i**, The total number of nose pokes under the PR2 task (n = 5 mice for each group, two-way repeated-measures ANOVA, Tukey's multiple comparisons test). **j**, The breakpoints in the PR2 task (n = 5 mice for each group, two-tailed unpaired t-test). **k**, Scheme of the free-reward consumption of a 10% sucrose paradigm. **l**, Raster plots of sugar licks of individual mice. Green shades represent 5-s duration of 532 nm laser photoinhibition. **m**, The average number of sugar licks (n = 18 mice, two-tailed unpaired t-test). Data are presented as the mean ± SEM. *P* values are indicated in all panels. Source data are provided as a Source Data File.

Supplementary Fig. 8: Expression of DIO-hChR2-EYFP in the LPAG.

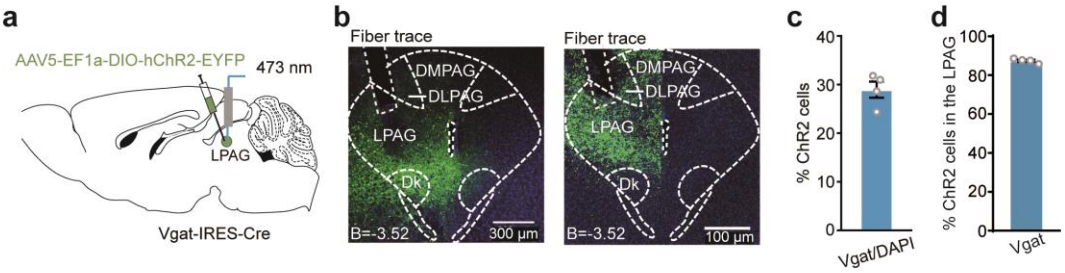

a, Scheme for photoactivation of LPAG^{Vgat} neurons. **b**, Histology of injection sites and fiber traces in the LPAG. Scale bars, 300 μm and 100 μm. **c**, Percentage of LPAG$^{Vgat\text{-}ChR2}$/LPAG^{DAPI} neurons (n = 4 mice). **d**, Percentage of Vgat+ neurons by ChR2 infected spread localized to the LPAG (n = 4 mice). Data are shown as the mean ± s.e.m.

Supplementary Fig. 9: Photoactivation of LPAG^{Vglut2} neurons induces defensive behaviors.

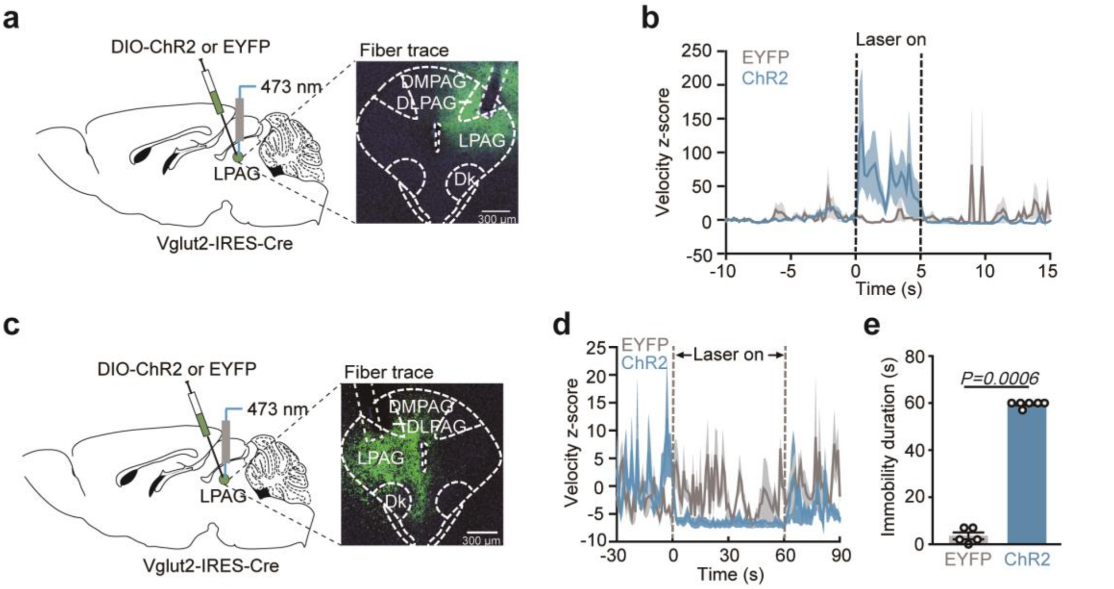

a, Left, scheme of virus injection and fiber implantation. Right, histology of injection site and fiber trace in the DL/LPAG. Scale bar, 300 μm. **b**, Photoactivation of DL/LPAG^{Vglut2} neurons induced flight responses (n = 4 mice for each group). **c**, Scheme of photoactivation of LPAG^{Vglut2} neurons. **d**, Z-score of velocity in ChR2 and EYFP groups. **e**, Photoactivation of LPAG^{Vglut2} neurons increased immobility duration (two-sided Mann Whitney U test). LPAG$^{Vgat\text{-}EYFP}$ mice, n = 5; LPAG$^{Vgat\text{-}ChR2}$ mice, n = 6. Shaded areas represent SEM. Data are presented as the mean ± SEM. *P* values are indicated in all panels. Source data are provided as a Source Data File.

Supplementary Fig. 10: The activity of LPAG^{Vglut2} neurons correlates to jaw muscle activity during predatory attack.

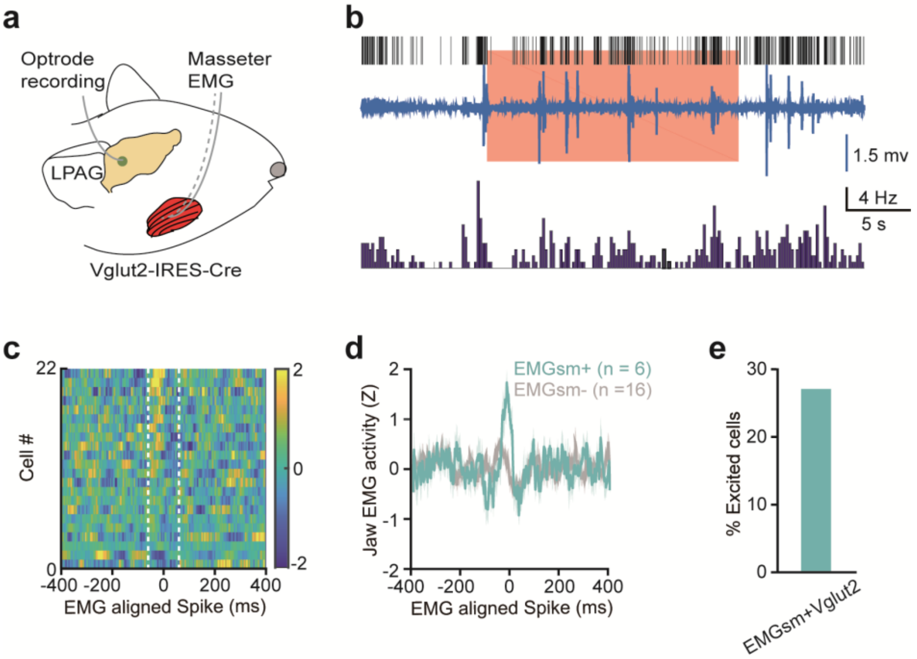

a, Scheme of simultaneous recordings of jaw muscle EMG and LPAG^{Vglut2} neurons during the attack phase of predatory hunting. **b**, An example of EMG (middle) and activity from simultaneously LPAG^{Vglut2} neuron (top and bottom) during the attack phase. The red shade represents the attack phase. **c**, Heatmap of EMG-aligned spikes recorded from LPAG^{Vglut2} neurons (n = 22 units). **d**, Normalized activity of EMG stimulus-related (EMGsm+), and -unrelated (EMGsm-) LPAG^{Vglut2} neurons during a predatory attack. Shaded areas represent SEM. **e**, Percentage of excited EMGsm+ LPAG^{Vglut2} neurons (n = 6 units) during a predatory attack.

Supplementary Fig. 11: Connectivity of CeA/LH/ZI-LPAG pathway.

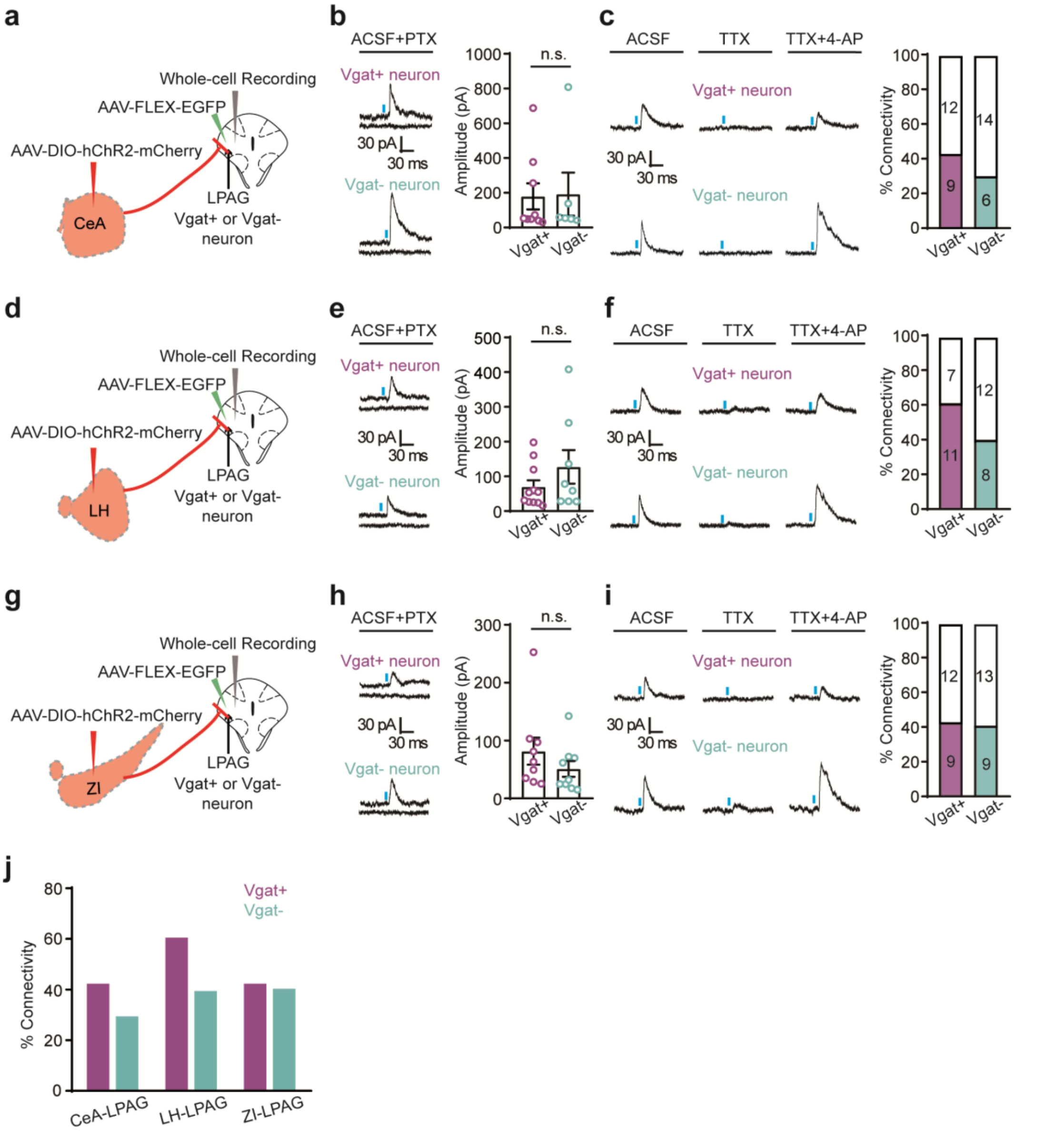

a-c, Connectivity of CeA-LPAG pathway. **a**, Experimental scheme. **b**, Example traces (left, by application of PTX) and quantitative analyses (right) of the amplitude of light-evoked IPSCs from the LPAG^{Vgat+} (n = 9) or LPAG^{Vgat-} (n = 6) neuron (two-tailed unpaired t-test, $P > 0.05$, n.s., no significance). **c**, Example traces (left, by application of TTX and 4-AP) and connection probabilities (right) of CeA-LPAG pathway. **d-f**, Connectivity of LH-LPAG pathway. **d**, Experimental scheme. **e**, Example traces (left, by application of PTX) and quantitative analyses (right) of the amplitude of light-evoked IPSCs from the LPAG^{Vgat+} (n = 11) or LPAG^{Vgat-} (n = 8) neuron (two-tailed unpaired t-test, $P > 0.05$, n.s., no significance). **f**, Example traces (left, by application of TTX and 4-AP) and connection probabilities (right) of LH-LPAG pathway. **g-i**, Connectivity of ZI-LPAG pathway. **g**, Experimental scheme. **h**, Example traces (left, by application of PTX) and quantitative analyses (right) of the amplitude of light-evoked IPSCs from the LPAG^{Vgat+} (n = 9) or LPAG^{Vgat-} (n = 9)

neuron (two-tailed unpaired t-test, $P > 0.05$, n.s., no significance). **i**, Example traces (left, by application of TTX and 4-AP) and connection probabilities (right) of ZI-LPAG pathway. **j**, Connection probabilities of CeA/LH/ZI-LPAG pathway. Data are presented as the mean ± SEM. Source data are provided as a Source Data File.

Supplementary Fig. 12: Anatomical quantification of LPAG-projecting CeA/LH/ZI neurons.

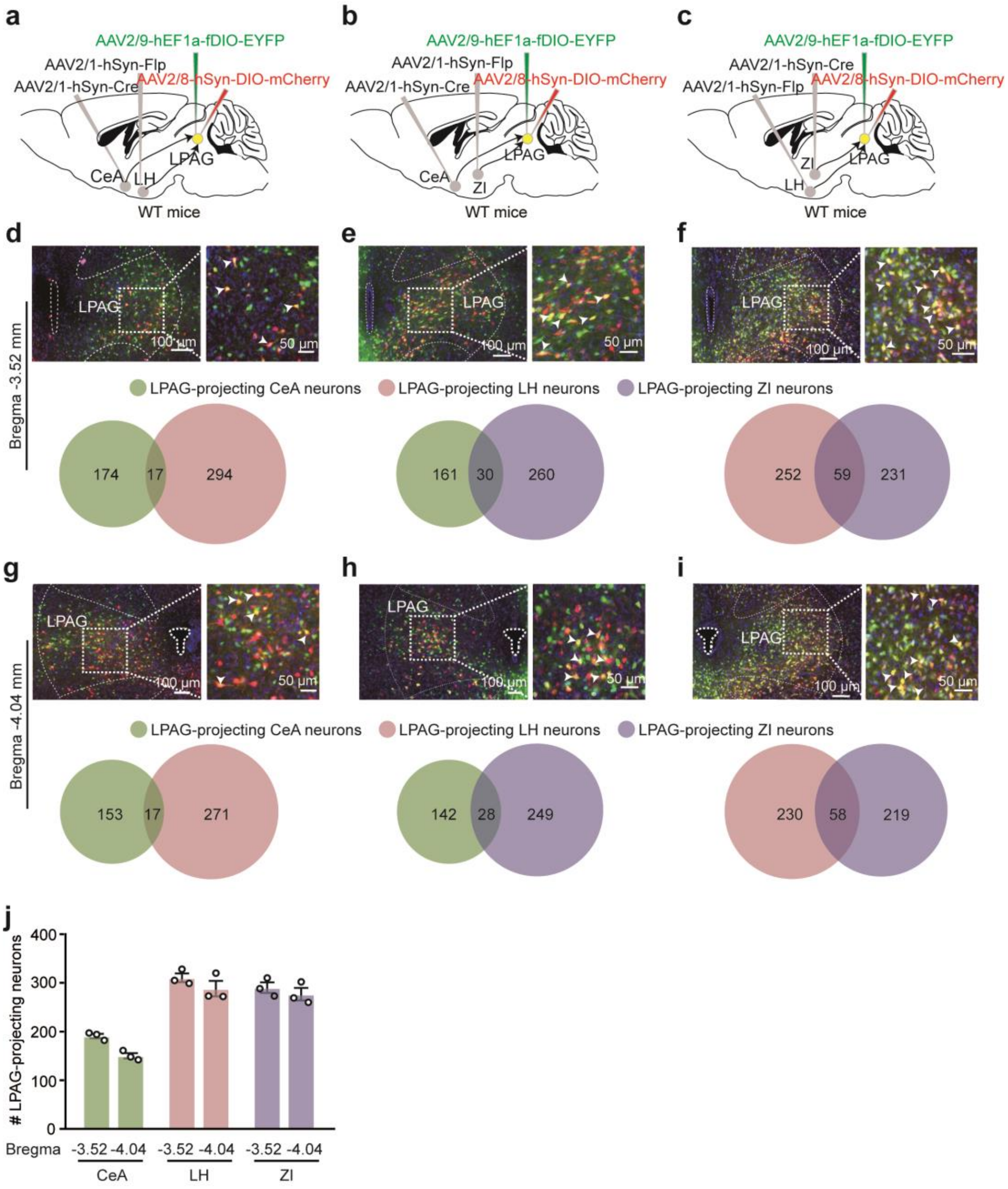

a-c, Experimental scheme of quantifying LPAG-projecting CeA/LH/ZI neurons. **d**, Top, images of LPAG-projecting CeA (red)/LH (green) neurons in WT mice. Scale bars, 100 μm and 50 μm. Bottom, the overlap between LPAG-projecting CeA neurons (red) and LPAG-projecting LH neurons (green) (n = 3 mice for each group). **e**, Top, images of LPAG-projecting CeA (red)/ZI (green) neurons in WT mice. Scale bars, 100 μm and 50 μm. Bottom, the overlap between LPAG-projecting CeA (red) neurons and LPAG-projecting ZI (green) neurons (n = 3 mice for each group). **f**, Top, images of LPAG-projecting LH (green)/ZI (red) neurons in WT mice. Scale bars, 100 μm and 50 μm. Bottom, the overlap between LPAG-projecting LH neurons (green) and LPAG-projecting ZI (red) neurons (n = 3 mice for each group). The bregma in **d-f** was -3.52 mm. **g**, Top, images of LPAG-projecting CeA (red)/LH (green) neurons in WT mice. Scale bars, 100 μm and 50 μm. Bottom, the overlap

between LPAG-projecting CeA neurons (red) and LPAG-projecting LH neurons (green) (n = 3 mice for each group). **h**, Top, images of LPAG-projecting CeA (red)/ZI (green) neurons in WT mice. Scale bars, 100 μm and 50 μm. Bottom, the overlap between LPAG-projecting CeA (red) neurons and LPAG-projecting ZI (green) neurons (n = 3 mice for each group). **i**, Top, images of LPAG-projecting LH (green)/ZI (red) neurons in WT mice. Scale bars, 100 μm and 50 μm. Bottom, the overlap between LPAG-projecting LH neurons (green) and LPAG-projecting ZI (red) neurons (n = 3 mice for each group). The bregma in **g-i** was -4.04 mm. White arrows in **d-i** represented overlap neurons. **j**, Quantification of LPAG-projecting CeA/LH/ZI neurons (n = 3 mice for each group). Data are presented as the mean ± SEM. Source data are provided as a Source Data File.

Supplementary Fig. 13: Phenotypes after ablation of distinct GABAergic inputs to the LPAG.

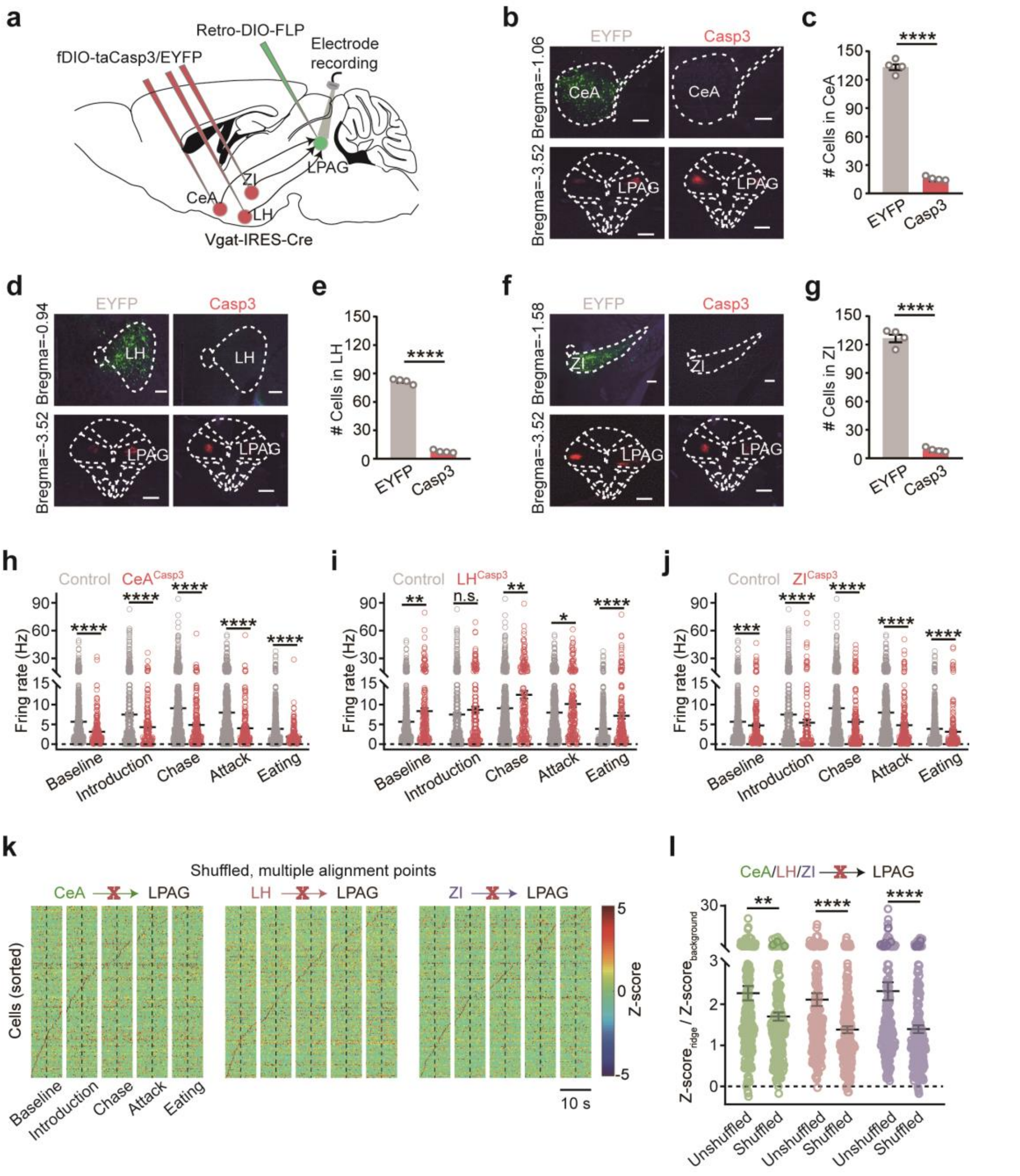

a, Experimental scheme of *in vivo* single-unit recordings in the LPAG after ablation of GABAergic neurons from the CeA, the LH, and the ZI, respectively. **b**, Top, images of LPAG-projecting GABAergic neurons from the CeA in Vgat-Cre:: EYFP (left) and Vgat-Cre:: Caspase3 & EYFP (right) mice. Bottom, injection sites of AAV-retro-DIO-Flp in the LPAG; CTB555 as an indicator. Scale bars, 200 μm. **c**, Quantification of the effects of caspase lesions on LPAG-projecting GABAergic neurons from the CeA (n = 4 mice for each group, two-tailed unpaired t-test, $P < 0.0001$). **d**, Top, images of LPAG-projecting GABAergic neurons from the LH in Vgat-Cre::

EYFP (left) and Vgat-Cre:: Caspase3 & EYFP (right) mice. Bottom, injection sites of AAV-retro-DIO-Flp in the LPAG; CTB555 as an indicator. Scale bars, 200 µm. **e**, Quantification of the effects of caspase lesions on LPAG-projecting GABAergic neurons from the LH (n = 4 mice for each group, two-tailed unpaired t-test, $P < 0.0001$). **f**, Top, images of LPAG-projecting GABAergic neurons from the ZI in Vgat-Cre:: EYFP (left) and Vgat-Cre:: Caspase3 & EYFP (right) mice. Bottom, injection sites of AAV-retro-DIO-Flp in the LPAG, CTB555 as an indicator. Scale bars, 200 µm. **g**, Quantification of the effects of caspase lesions on LPAG-projecting GABAergic neurons from the ZI (n = 4 mice for each group, two-tailed unpaired t-test, $P < 0.0001$). **h**, Firing rates of neurons recorded during different predatory phases in the control and mice after ablation of LPAG-projecting GABAergic neurons from the CeA (n = 203 units, two-sided Wilcoxon rank-sum test, $P < 0.0001$). **i**, Firing rates of neurons recorded during different predatory phases in the control and mice after ablation of LPAG-projecting GABAergic neurons from the LH (n = 199 units, two-sided Wilcoxon rank-sum test, $P = 0.055$, $P = 0.1673$, $P = 0.0023$, $P = 0.0455$, $P < 0.0001$). **j**, Firing rates of neurons recorded during different predatory phases in the control and mice after ablation of LPAG-projecting GABAergic neurons from the ZI (n = 206 units, two-sided Wilcoxon rank-sum test, $P = 0.0003$, $P < 0.0001$). **k**, Normalized responses of all recorded neurons during predatory hunting, sorted by their peak of responses. Shuffled datasets from the same set of neurons as in Fig. 5**k**, 5**l**, and 5**m**, respectively (based on 1000 shuffles). Dotted lines represent the timepoints of put-in of crickets, chase onset, first attack, and eating onset, respectively. **l**, Comparison of ridge-to-background Z-score ratio between unshuffled and shuffled datas after ablation of LPAG-projecting GABAergic neurons from the CeA/LH/ZI, respectively (two-sided Wilcoxon signed-rank test, $P = 0.0003$, $P < 0.0001$, $P < 0.0001$). The ridge was defined as the mean z-score in 5 bins surrounding the peak value, and the background was defined as the mean z-score in all data points. The ridge-to-background ratio provides a measure of how selective the activity of the cell was during hunting. Data are presented as the mean ± SEM. Source data are provided as a Source Data File.

Supplementary Fig. 14: Behavioral phenotypes after ablation of LPAG-projecting GABAergic neurons from the CeA/LH/ZI.

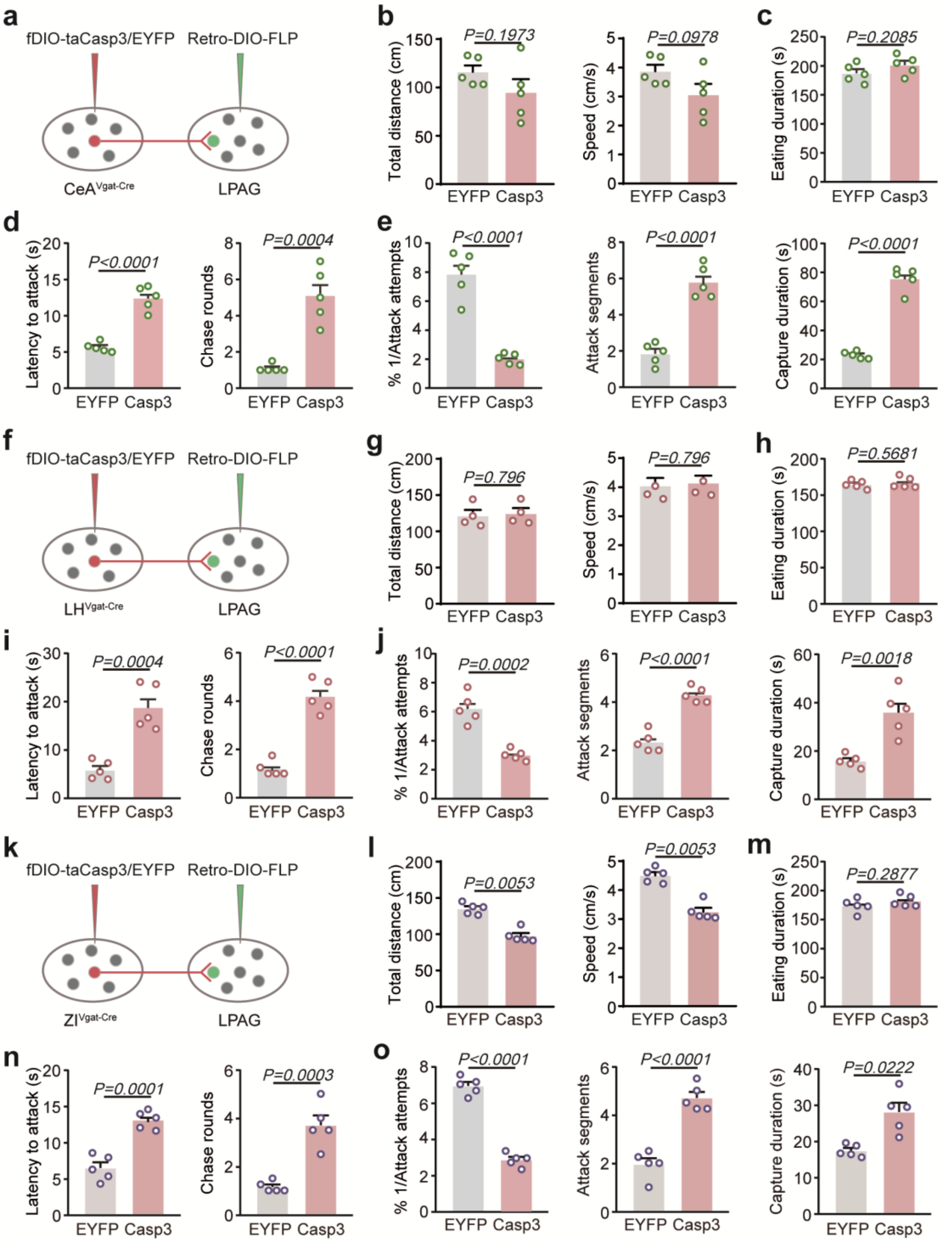

a-e, Ablation of LPAG-projecting GABAergic neurons from the CeA. **a**, Experimental scheme. **b**, The open field test. Total distance (left), average velocity (right) (n = 5 mice for each group, two-tailed unpaired t-test). **c**, The effect on the eating phase (n = 5 mice for each group, two-tailed unpaired t-test). **d**, The effect on the chase phase (n = 5 mice for each group, two-tailed unpaired t-test). **e**, The effect on the attack phase (n = 5 mice for each group, two-tailed unpaired t-test). **f-j**, Ablation of LPAG-projecting GABAergic neurons from the LH. **f**, Experimental scheme. **g**, The

open field test. Total distance (left), average velocity (right) (n = 4 mice for each group, two-tailed unpaired t-test). **h**, The effect on the eating phase (n = 5 mice for each group, two-tailed unpaired t-test). **i**, The effect on the chase phase (n = 5 mice for each group, two-tailed unpaired t-test). **j**, The effect on the attack phase (n = 5 mice for each group, two-tailed unpaired t-test). **k-o**, Ablation of LPAG-projecting GABAergic neurons from the ZI. **k**, Experimental scheme. **l**, The open field test. Total distance (left), average velocity (right) (n = 5 mice for each group, two-tailed unpaired t-test). **m**, The effect on the eating phase (n = 5 mice for each group, two-tailed unpaired t-test). **n**, The effect on the chase phase (n = 5 mice for each group, two-tailed unpaired t-test). **o**, The effect on the attack phase (n = 5 mice for each group, two-tailed unpaired t-test). Data are presented as the mean ± SEM. *P* values are indicated in all panels. Source data are provided as a Source Data File.

Supplementary Table 1 Summary of statistical analyses

Figure	Sample size (n)	Statistical test	P values
1d	618 units	Wilcoxon signed-rank test	Introduction vs. Baseline: $P < 0.0001^{****}$
			Chase vs. Baseline: $P < 0.0001^{****}$
			Attack vs. Baseline: $P < 0.0001^{****}$
			Eating vs. Baseline: $P < 0.0001^{****}$
1j	618 units	Wilcoxon signed-rank test	$P < 0.0001^{****}$
3c	ArchT: 6 mice	Two-tailed paired t-test	ArchT: $P = 0.0024^{**}$
	EYFP: 6 mice		EYFP: $P = 0.9353$ n.s.
3d	ArchT: 6 mice	Two-tailed paired t test	ArchT: $P = 0.0018^{**}$
	EYFP: 6 mice		EYFP: $P = 0.6109$ n.s.
3e	ArchT: 6 mice	Two-tailed paired t-test	ArchT: $P = 0.6796$ n.s.
	EYFP: 6 mice		EYFP: $P = 0.5673$ n.s.
3f	ArchT: 6 mice	Two-tailed paired t-test	ArchT: $P = 0.004^{**}$
	EYFP: 6 mice		EYFP: $P = 0.5248$ n.s.
3g	ArchT: 6 mice	Two-tailed paired t-test	ArchT: $P = 0.0011^{**}$
	EYFP: 6 mice		EYFP: $P = 0.8357$ n.s.
3h	ArchT: 6 mice	Two-tailed paired t-test	ArchT: $P = 0.0012^{**}$
	EYFP: 6 mice		EYFP: $P > 0.9999$ n.s.
4c	ArchT: 6 mice	Two-tailed paired t-test	ArchT: $P = 0.9918$ n.s.
	EYFP: 5 mice		EYFP: $P = 0.6478$ n.s.
4d	ArchT: 6 mice	Two-tailed paired t-test	ArchT: $P = 0.4444$ n.s.
	EYFP: 5 mice		EYFP: $P = 0.3739$ n.s.
4e	ArchT: 6 mice	Two-tailed paired t-test	ArchT: $P = 0.3492$ n.s.
	EYFP: 5 mice		EYFP: $P = 0.1818$ n.s.
4f	ArchT: 6 mice	Two-tailed paired t-test	ArchT: $P = 0.0005^{***}$
	EYFP: 5 mice		EYFP: $P = 0.2187$ n.s.
4g	ArchT: 6 mice	Two-tailed paired t-test	ArchT: $P = 0.0198^{**}$
	EYFP: 5 mice		EYFP: $P = 0.2855$ n.s.
4h	ArchT: 6 mice	Two-tailed paired t-test	ArchT: $P = 0.0412^{*}$
	EYFP: 5 mice		EYFP: $P = 0.5673$ n.s.
4i	ArchT: 6 mice	Two-tailed paired t-test	ArchT: $P = 0.1019$ n.s.
	EYFP: 5 mice		EYFP: $P > 0.9999$ n.s.
5g	Control: 618 units	Chi-square test	Type I: control vs. casp3 $P = 0.0352^{*}$
	Casp3: 203 units		Type IV: control vs. casp3 $P < 0.0001^{****}$
			Type III: control vs. casp3 $P = 0.0528$ n.s.
			Type VI: control vs. casp3 $P < 0.0001^{****}$

Figure	Sample size (n)	Statistical test	P values
			Type II: control vs. casp3 *P* = 0.0005***
			Type V: control vs. casp3 *P* < 0.0001****
			Type VII: control vs. casp3 *P* = 0.0553 n.s.
5h	Control: 618 units	Chi-square test	Type I: control vs. casp3 *P* = 0.2116 n.s
	Casp3: 199 units		Type IV: control vs. casp3 *P* = 0.0023**
			Type III: control vs. casp3 *P* = 0.2413 n.s.
			Type VI: control vs. casp3 *P*= 0.1659 n.s.
			Type II: control vs. casp3 *P* = 0.0092**
			Type V: control vs. casp3 *P* < 0.0001****
			Type VII: control vs. casp3 *P* = 0.1868 n.s.
5i	Control: 618 units	Chi-square test	Type I: control vs. casp3 *P* = 0.0342*
	Casp3: 206 units		Type IV: control vs. casp3 *P* = 0.1173 n.s.
			Type III: control vs. casp3 *P* = 0.0515 n.s.
			Type VI: control vs. casp3 *P* < 0.0001****
			Type II: control vs. casp3 *P* = 0.0088**
			Type V: control vs. casp3 *P* = 0.1356 n.s.
			Type VII: control vs. casp3 *P* = 0.008**
6b	30 mice	Two-way repeated measures ANOVA	Region: F (1, 24) = 104.8, *P* < 0.0001****
			Virus: F (2, 24) = 5.381, *P* = 0.0117*
		factor one: region (CeA, LH, ZI)	Interaction: F (2, 24) = 5.913, *P* = 0.0082**
		factor two: virus (EYFP, Casp3)	multiple comparisons:
		Tukey's multiple comparisons test	EYFP: CeA vs. LH: *P* = 0.991 n.s.
			CeA vs. ZI: *P* = 0.854 n.s.
			LH vs. ZI: *P* = 0.9123 n.s.
			Casp3: CeA vs. LH: *P* = 0.0007***
			CeA vs. ZI: *P* = 0.9041 n.s.
			LH vs. ZI: *P* = 0.0021**
6c	30 mice	Two-way repeated measures ANOVA	Region: F (1, 24) = 120.4, *P* < 0.0001****
			Virus: F (2, 24) = 1.875, *P* = 0.1752 n.s.
		factor one: region (CeA, LH, ZI)	Interaction: F (2, 24) = 2.237, *P* = 0.1285 n.s.
		factor two: virus (EYFP, Casp3)	multiple comparisons:
		Tukey's multiple comparisons test	EYFP: CeA vs. LH: *P* > 0.9999 n.s.
			CeA vs. ZI: *P* > 0.9999 n.s.
			LH vs. ZI: *P* > 0.9999 n.s.
			Casp3: CeA vs. LH: *P* = 0.2405 n.s.
			CeA vs. ZI: *P* = 0.0284*

Figure	Sample size (n)	Statistical test	P values
			LH vs. ZI: $P = 0.9919$ n.s.
6d	30 mice	Two-way repeated measures ANOVA<br>factor one: region (CeA, LH, ZI)<br>factor two: virus (EYFP, Casp3)<br>Tukey's multiple comparisons test	Region: F (1, 24) = 162.9, $P < 0.0001^{****}$<br>Virus: F (2, 24) = 0.325, $P = 0.7256$ n.s.<br>Interaction: F (2, 24) = 3.483, $P = 0.047^{*}$<br>multiple comparisons:<br>EYFP: CeA vs. LH: $P = 0.1904$ n.s.<br>CeA vs. ZI: $P = 0.833$ n.s.<br>LH vs. ZI: $P = 0.4512$ n.s.<br>Casp3: CeA vs. LH: $P = 0.1576$ n.s.<br>CeA vs. ZI: $P = 0.2587$ n.s.<br>LH vs. ZI: $P = 0.9533$ n.s.
6e	30 mice	Two-way repeated measures ANOVA<br>factor one: region (CeA, LH, ZI)<br>factor two: virus (EYFP, Casp3)<br>Tukey's multiple comparisons test	Region: F (1, 24) = 179.2, $P < 0.0001^{****}$<br>Virus: F (2, 24) = 0.2.395, $P = 0.1126$ n.s.<br>Interaction: F (2, 24) = 7.281, $P = 0.0034^{**}$<br>multiple comparisons:<br>EYFP: CeA vs. LH: $P = 0.3874$ n.s.<br>CeA vs. ZI: $P = 0.9612$ n.s.<br>LH vs. ZI: $P = 0.54$ n.s.<br>Casp3: CeA vs. LH: $P = 0.0014^{**}$<br>CeA vs. ZI: $P = 0.0185^{*}$<br>LH vs. ZI: $P = 0.54$ n.s.
6f	30 mice	Two-way repeated measures ANOVA<br>factor one: region (CeA, LH, ZI)<br>factor two: virus (EYFP, Casp3)<br>Tukey's multiple comparisons test	Region: F (1, 24) = 177.4, $P < 0.0001^{****}$<br>Virus: F (2, 24) = 68.33, $P < 0.0001^{****}$<br>Interaction: F (2, 24) = 39.16, $P < 0.0001^{****}$.<br>multiple comparisons:<br>EYFP: CeA vs. LH: $P = 0.117$ n.s.<br>CeA vs. ZI: $P = 0.2629$ n.s.<br>LH vs. ZI: $P = 0.8872$ n.s.<br>Casp3: CeA vs. LH: $P < 0.0001^{****}$<br>CeA vs. ZI: $P < 0.0001^{****}$<br>LH vs. ZI: $P = 0.052$ n.s.
6g	30 mice	Two-way repeated measures ANOVA<br>factor one: region (CeA, LH, ZI)<br>factor two: virus (EYFP, Casp3)<br>Tukey's multiple comparisons	Region: F (1, 24) = 5.271, $P = 0.0307^{*}$<br>Virus: F (2, 24) = 5.261, $P = 0.0127^{*}$<br>Interaction: F (2, 24) = 0.1265, $P = 0.8817$<br>multiple comparisons:<br>EYFP: CeA vs. LH: $P = 0.2131$ n.s.

Figure	Sample size (n)	Statistical test	P values
		test	CeA vs. ZI: *P* = 0.227 n.s.
			LH vs. ZI: *P* = 0.992 n.s.
			Casp3: CeA vs. LH: *P* = 0.1128 n.s.
			CeA vs. ZI: *P* = 0.0602 n.s.
			LH vs. ZI: *P* = 0.9465 n.s.
