# Supplementary material for: Periaqueductal gray neurons encode the sequential motor program in hunting behavior of mice
Source: Nat Commun. 2021 Nov 11;12:6523. doi: 10.1038/s41467-021-26852-1 (PMC8586038; doi:10.1038/s41467-021-26852-1)
Supplement: Supplementary file 3 — Description of Additional Supplementary Files [file 41467_2021_26852_MOESM3_ESM.pdf]

Description of Additional Supplementary Files

File Name: Supplementary Movie 1
Description: This movie shows that the predatory motor sequence was blocked when the cricket was removed during the chase phase.

File Name: Supplementary Movie 2
Description: This movie shows that the predatory motor sequence was blocked when the cricket was removed during the attack phase.

File Name: Supplementary Movie 3
Description: This movie shows the spontaneous movement of a CeA-LPAG^{Casp3} mouse.

File Name: Supplementary Movie 4
Description: This movie shows the spontaneous movement of a CeA-LPAG^{EYFP} mouse.

File Name: Supplementary Movie 5
Description: This movie shows the spontaneous movement of an LH-LPAG^{Casp3} mouse.

File Name: Supplementary Movie 6
Description: This movie shows the spontaneous movement of an LH-LPAG^{EYFP} mouse.

File Name: Supplementary Movie 7
Description: This movie shows the spontaneous movement of a ZI-LPAG^{Casp3} mouse.

File Name: Supplementary Movie 8
Description: This movie shows the spontaneous movement of a ZI-LPAG^{EYFP} mouse.
