# Supplementary material for: Periaqueductal gray neurons encode the sequential motor program in hunting behavior of mice
Source: Nat Commun. 2021 Nov 11;12:6523. doi: 10.1038/s41467-021-26852-1 (PMC8586038; doi:10.1038/s41467-021-26852-1)
Supplement: Supplementary file 12 — Reporting Summary [file 41467_2021_26852_MOESM12_ESM.pdf]

nature portfolio

Corresponding author(s): Wei L. Shen, Haohong Li

Last updated by author(s): September 28, 2021

Reporting Summary

Nature Portfolio wishes to improve the reproducibility of the work that we publish. This form provides structure for consistency and transparency in reporting. For further information on Nature Portfolio policies, see our Editorial Policies and the Editorial Policy Checklist.

Statistics

For all statistical analyses, confirm that the following items are present in the figure legend, table legend, main text, or Methods section.

n/a	Confirmed	
☐	☒	The exact sample size (*n*) for each experimental group/condition, given as a discrete number and unit of measurement
☐	☒	A statement on whether measurements were taken from distinct samples or whether the same sample was measured repeatedly
☐	☒	The statistical test(s) used AND whether they are one- or two-sided<br>*Only common tests should be described solely by name; describe more complex techniques in the Methods section.*
☒	☐	A description of all covariates tested
☐	☒	A description of any assumptions or corrections, such as tests of normality and adjustment for multiple comparisons
☐	☒	A full description of the statistical parameters including central tendency (e.g. means) or other basic estimates (e.g. regression coefficient) AND variation (e.g. standard deviation) or associated estimates of uncertainty (e.g. confidence intervals)
☐	☒	For null hypothesis testing, the test statistic (e.g. *F*, *t*, *r*) with confidence intervals, effect sizes, degrees of freedom and *P* value noted<br>*Give P values as exact values whenever suitable.*
☒	☐	For Bayesian analysis, information on the choice of priors and Markov chain Monte Carlo settings
☒	☐	For hierarchical and complex designs, identification of the appropriate level for tests and full reporting of outcomes
☐	☒	Estimates of effect sizes (e.g. Cohen's *d*, Pearson's *r*), indicating how they were calculated

Our web collection on statistics for biologists contains articles on many of the points above.

Software and code

Policy information about availability of computer code

Data collection	For in vivo single-unit recordings, electrical signals were amplified (× 200 000 gain) and digitized at 30 kHz by Open Ephys Recording System (https://open-ephys.org/). Behavioral videos were recorded using commercial camera (Logitech, C1000e, 60 fps). For EMG recording, signals were recorded using a Microelectrode AC Amplifier Model 1800 (A-M Systems, USA), filtered (10–500 Hz) and digitized at 1000 Hz by Spike Hound software Gus K. Lott, III (http://spikehound.sourceforge.net/). Whole-cell patch-clamp recordings were performed with a MultiClamp 700B amplifier (Molecular Devices, CA, USA) connected to a Digidata 1440A interface (Axon, USA). Data were analyzed using Clampfit 10.0 software.
Data analysis	For cricket hunting experiments, behavioral events were manually analyzed based on recorded videos. In vivo single-unit recording data was analyzed using MATLAB R2014b (The Mathworks, Inc., Natick, Massachusetts, USA). Single-unit spikes sorting was performed using the toolbox MClust-4.4 (a MATLAB based free-ware spike sorting toolset developed by A David Redish). In intracranial self-stimulation (ICSS) experiments, nose pokes were analyzed using LabState ver1.0 (AniLab, China). For RTPP, videos were analyzed using idTracker.ai v3 (https://www.idtracker.es). All data was replotted and statistical analyzed using GraphPad Prism 7 (GraphPad, USA). We have uploaded our custom code to: https://github.com/xinkuaninGit/PAG_hunting.git.

For manuscripts utilizing custom algorithms or software that are central to the research but not yet described in published literature, software must be made available to editors and reviewers. We strongly encourage code deposition in a community repository (e.g. GitHub). See the Nature Portfolio guidelines for submitting code & software for further information.

Data

Policy information about availability of data

All manuscripts must include a data availability statement. This statement should provide the following information, where applicable:

- Accession codes, unique identifiers, or web links for publicly available datasets
- A description of any restrictions on data availability
- For clinical datasets or third party data, please ensure that the statement adheres to our policy

The raw data for Figs. 1-6 and Supplementary Fig. 1-14 are provided in an Excel file as Source Data. Source data are provided with this paper.

Field-specific reporting

Please select the one below that is the best fit for your research. If you are not sure, read the appropriate sections before making your selection.

☒ Life sciences ☐ Behavioural & social sciences ☐ Ecological, evolutionary & environmental sciences

For a reference copy of the document with all sections, see nature.com/documents/nr-reporting-summary-flat.pdf

Life sciences study design

All studies must disclose on these points even when the disclosure is negative.

Sample size	No sample-size calculation was performed, but our study used similar sample sizes as previous studies (PMID: 31127258; PMID: 31127260; PMID: 29398361). Wilcox signed-rank test, Wilcoxon rank-sum test, two-tailed paired and unpaired t-test, one-way ANOVA, two-way ANOVA, Mann Whitney U test, Kruskal-Wallis test were used to determine the significance (see details in the relevant figure legends and the Supplemental Table 1: Summary of statistical analyses).
Data exclusions	For all experiments, mice with missed viral injections or implantation targets, as described by brain atlas, were not included in experimental analyses. For in vivo single-unit recordings, manually spikes sorting was based on previous study (PMID: 29141212). Neurons with isolation distance (< 20) and L-ratio (> 0.1) were excluded, as they are considered noise. A unit that inter-spike intervals spikes count in 2 ms were less than 1% was contained in the analysis. A cross-correlation comparison was performed to avoid repeat units in a session.
Replication	The experiments were repeated for 3-7 times in independent mice, depending on different experiments. Data were analyzed using a double-blind method.
Randomization	Randomization was not required because this study did not allocate different mouse experimental groups.
Blinding	All investigators were blinded to group allocation during data collection and analysis.

Reporting for specific materials, systems and methods

We require information from authors about some types of materials, experimental systems and methods used in many studies. Here, indicate whether each material, system or method listed is relevant to your study. If you are not sure if a list item applies to your research, read the appropriate section before selecting a response.

Materials & experimental systems

n/a	Involved in the study	
☒	☐	Antibodies
☒	☐	Eukaryotic cell lines
☒	☐	Palaeontology and archaeology
☐	☒	Animals and other organisms
☒	☐	Human research participants
☒	☐	Clinical data
☒	☐	Dual use research of concern

Methods

n/a	Involved in the study	
☒	☐	ChIP-seq
☒	☐	Flow cytometry
☒	☐	MRI-based neuroimaging

Animals and other organisms

Policy information about studies involving animals; ARRIVE guidelines recommended for reporting animal research

Laboratory animals	Experimental subjects were adult male mice (8–16 weeks old) and crickets (oilgourd, 2–2.5 cm in size). The Vgat-ChR2 mice were obtained from Dr. Josh Huang Lab (Cold Spring Harbor Laboratory, Cold Spring Harbor, NY, USA). The Vglut2-IRES-Cre, Vgat-IRES-Cre and Ai-32 mice (Jackson Laboratories stock numbers: 028863, 028862, and 024109) were purchased from the Jackson Laboratory.

	C57BL/6J mice were from Beijing HFK Bioscience co., LTD. All mice were group-housed and bred under a constant temperature (22 ± 2℃), humidity (40%–60%) and 12-hr light/dark cycle (7:00 am to 19:00 pm).
Wild animals	The study did not involve wild animals.
Field-collected samples	This study did not involve samples collected from the field.
Ethics oversight	All experimental procedures were approved by the Hubei Provincial Animal Care and Use Committee and complied with the experimental guidelines of the Animal Experimentation Ethics Committee of Huazhong University of Science and Technology, ShanghaiTech University, and Shanghai Biomodel Organism Co., China.

Note that full information on the approval of the study protocol must also be provided in the manuscript.
